# Analysis of patients with colorectal cancer shows a specific increase in serum anti-ING1 autoantibody levels

**DOI:** 10.1186/s12885-023-10845-y

**Published:** 2023-04-18

**Authors:** Takahiro Arasawa, Takaki Hiwasa, Akiko Kagaya, Tetsuro Maruyama, Masaya Uesato, Masayuki Kano, Sohei Kobayashi, Hirotaka Takizawa, Katsuro Iwase, Fumio Nomura, Kazuyuki Matsushita, Hisahiro Matsubara

**Affiliations:** 1 Department of Frontier Surgery, Graduate School of Medicine, Chiba University, Chiba, 260-8670 Japan; 2 Department of Biochemistry and Genetics, Graduate School of Medicine, Chiba University, Chiba, 260-8670 Japan; 3 Department of Neurological Surgery, Graduate School of Medicine, Chiba University, Inohana 1-8-1, Chuo-Ku, Chiba, 260-8670 Japan; 4 Clinical Research Center, Chiba University Hospital, Chiba, 260-8677 Japan; 5 Department of Laboratory Medicine & Division of Clinical Genetics, Chiba University Hospital, Chiba, 260-8677 Japan; 6 Department of Medical Technology & Sciences, School of Health Sciences at Narita, International University of Health and Welfare, Chiba, 286-8686 Japan; 7 Port Square Kashiwado Clinic, Kashiwado Memorial Foundation, Chiba, 260-0025 Japan; 8 Division of Clinical Genetics, Chiba Foundation for Health Promotion & Disease Prevention, Chiba, 261-0002 Japan

**Keywords:** Colorectal cancer, Protein array analysis, Antibody, Tumor biomarker, Inhibitor of growth protein 1

## Abstract

Colorectal cancer (CRC) is the third most prevalent cancer in the world, yet the sensitivity and specificity of biomarkers for CRC diagnosis are insufficient. In the present study, we performed a protein microarray screening method to identify antibody markers for CRC. Inhibitor of growth family 1 (ING1) was identified as a candidate tumor antigen for CRC using protein microarrays (ProtoArray). Subsequent amplified luminescence proximity homogeneous assay-linked immunosorbent assay using recombinant ING1 protein showed that the serum levels of anti-ING1 antibodies were increased not only in patients with CRC but also in those with esophageal cancer (EC), gastric cancer (GC), breast cancer (BrC), and pancreatic cancer (PC) compared with those of healthy donors (HDs). Antibodies against the ING1 amino acids between 239 and 253 were present at significantly higher levels in patients with CRC than in those with EC, GC, BrC, or PC. Anti-ING1 antibody levels were significantly higher in the patients with CRC at any stages than in the HDs. Immunohistochemical staining revealed higher expression of ING1 protein in CRC cells than in the adjacent normal tissues. In luciferase reporter assays using a CRC cell line, ING1 augmented p53-mediated NOXA promoter activity but attenuated p53-stimulated Bax, p21, and PUMA promoter activities. Consequently, serum anti-ING1 antibodies can be used for sensitive and specific diagnoses of CRC.

**Supplementary Information:**

The online version contains supplementary material available at 10.1186/s12885-023-10845-y.

## Background

Colorectal cancer (CRC) is one of the most common cancers worldwide. Combined CRC occurrence in men and women makes it the third most prevalent cancer after lung and breast cancers, and the related deaths are the second most numerous after those due to lung cancer [1]. In its early stages, CRC is confined to mucosal or submucosal tissues, and endoscopic or surgical resection is associated with complete cure and high patient survival rates. CRC that has progressed at the time of diagnosis requires multidisciplinary treatments by surgical excision, chemotherapy, and radiation therapy, leading to reduced patient longevity and quality of life. Therefore, diagnostic technologies for early-stage CRC are eagerly sought.

Fecal occult blood tests are noninvasive and convenient but have low sensitivity and specificity for early-stage CRC [2], reflecting the useful qualities of serum tumor markers. Traditional CRC serum markers include carcinoembryonic antigen and carbohydrate antigen 19–9; however, these are not sufficiently detectable during early tumor development [3, 4]. Although serum p53 antibody tests have demonstrated efficacy in the detection of early-stage tumors [5, 6], few antibody markers have been clinically employed. Given that immunoglobulin G (IgG) antibodies are highly stable and reactive to specific antigens, the introduction of novel antibody markers could improve specificity and sensitivity.

Serological identification of antigens by recombinant cDNA expression cloning (SEREX) and protein microarray (ProtoArray) are comprehensive screening methods for identifying antigens recognized by serum IgG antibodies [7, 8]. By analyzing serum antibody levels, some novel antibody markers have been selected [9, 10]. For example, in our ProtoArray experiments, inhibitor of growth protein 1 (ING1) was identified as a candidate CRC antigen, and we further showed that serum anti-ING1 antibody (s-ING1-Ab) levels were significantly higher in patients with CRC than in healthy donors (HDs).

## Methods

### Sera from patients with cancer and healthy donors

The study protocol was approved by the ethics committee of the Graduate School of Medicine, Chiba University, and by the cooperating hospitals. We collected patient sera prior to treating cancers and after obtaining written informed consent from patients with CRC (*n* = 192), esophageal cancer (EC, *n* = 96), gastric cancer (GC, *n* = 96), breast cancer (BrC, *n* = 93), and pancreatic cancer (PC, *n* = 50) at Chiba University Hospital. CRC staging was classified according to the Japanese Classification of Colorectal, Appendiceal, and Anal Carcinoma, 8th edition. We also collected sera from HDs (*n* = 128) at the Port Square Kashiwado Clinic. Immediately after collection, we centrifuged the serum samples at 2000 g for 5 min and stored the supernatants at -80 °C until use. Repeated thawing and freezing of samples was avoided.

### Protein microarray screening

We performed the screening using the ProtoArray v5.1 human protein microarray system (Thermo Fisher Scientific, Waltham, MA), which comprises 9375 protein species. We employed Alexa Fluor 647-anti-human IgG detection reagent to quantify the fluorescence intensity which represented the serum IgG bound to immobilized proteins. Results were analyzed using the Prospector software (Thermo Fisher Scientific), and a cutoff for positivity was calculated for each protein using M-statistics. For both groups, the proportion of subjects with an immune response above the cutoff value was counted. The algorithm to select candidate antigens in our study determined that the antigen positivity rate in the sera of patients with CRC was more than 60%, and the positivity rate in the sera of HDs was 0% as described [8, 9].

### Reverse transcription polymerase chain reaction

We isolated total RNA from the DLD-1 CRC cell line using an AquaPure RNA Isolation Kit (Catalog number 732–6370, Bio-Rad, Hercules, CA). We performed reverse transcription with oligo(dT)_20_ primers (Thermo Fisher Scientific) using a ThermoScript reverse transcription polymerase chain reaction (PCR) System (Thermo Fisher Scientific) as described previously [7, 10].

We amplified a full-length cDNA insert of *ING1* by a PCR with ING1 sense (5′-TAGAATTCTTGAGTCCTGCCAACGGG-3′) and antisense (5′-TTCTCGAGCTACCTGTTGTAAGCCCT-3′) primers and PrimeSTAR GXL DNA Polymerase (Takara Bio, Kusatsu, Japan). We performed thermal cycling with an initial denaturation step at 98 °C for 1 min, followed by 30 cycles of denaturation at 98 °C for 10 s, annealing at 56 °C for 15 s, and extension at 68 °C for 2 min, with a final extension step at 68 °C for 3 min.

### Construction of expression plasmid vectors

We constructed expression plasmids for the glutathione-S-transferase (GST)-fusion protein as described previously [11, 12]. Briefly, PCR-amplified full-length cDNA inserts of *ING1* were digested with *EcoR*I and *Xho*I and were separated using agarose gel electrophoresis. We then recovered the cDNA fragments using a MinElute Gel Extraction Kit (Qiagen, Hilden, Germany), and ligated them into the pGEX-4 T-1 vector (GE Healthcare Life Sciences, Pittsburgh, PA) using a Ligation-Convenience Kit (Nippon Gene, Tokyo, Japan) after pre-digestion of the plasmid with *EcoR*I and *Xho*I.

### Purification of recombinant proteins

We transformed ECOS competent *Escherichia coli* JM-109 cells (Nippon Gene) with the recombinant plasmid pGEX-4 T-1-ING1 and cultured them for 3 h in 200-ml Luria broth containing 0.1 mM isopropyl β-D-thiogalactopyranoside (FUJIFILM Wako Pure Chemical, Osaka, Japan). We then harvested the cells, washed them with phosphate-buffered saline, and lysed them by sonication in Y-PER Yeast Protein Extraction Reagent (Thermo Fisher Scientific). We centrifuged the lysates at 15,000 g for 10 min at 4 °C and purified the GST-fused ING1 proteins in supernatants using affinity chromatography with Glutathione-Sepharose columns (GE Healthcare Life Sciences) according to the manufacturer's instructions. We finally concentrated the purified proteins with an Amicon Ultra-15 Centrifugal Filter Device (Merck KGaA, Millipore, Darmstadt, Germany) as described [12, 13].

### Immunohistochemical staining of ING1

We sectioned and then dewaxed paraffin-embedded CRC tumor tissues using graded alcohol and xylene. After antigen retrieval at 98 °C for 40 min in 10 mM citrate buffer (pH 6.0), we blocked endogenous peroxidase using 3% hydrogen peroxide in methanol for 30 min. We then washed all Sects. 3 times in wash buffer (S3006; DAKO, Carpinteria, CA) for 5 min each and then incubated them for 1 h with anti-human ING1 mouse monoclonal Ab (Clone 585,922; R&D Systems, Minneapolis, MN) (antibodies are shown in Additional file 1: Table S1) at a dilution of 1 to 200 at 37 °C for 60 min. Subsequently, we washed the Sects. 3 times with wash buffer (S3006; DAKO) for 5 min each and then incubated them with horseradish peroxidase-conjugated anti-rabbit/mouse secondary antibodies (EnVision™ Detection System: K5007; DAKO) at 37 °C for 60 min. We visualized the bound antibodies with the chromogen diaminobenzidine in 3% hydrogen peroxidase. Finally, we counterstained the sections with hematoxylin and dehydrated and mounted them on glass slides as described previously [9, 11].

### Western blot analysis

We electrophoresed GST and GST-ING1 proteins (0.3 μg per lane) on sodium dodecyl sulfate–polyacrylamide (11%) gels followed by western blotting using an anti-GST antibody (Catalog number 600–101-200, Rockland, Gilbertsville, PA), 2 randomly selected sera from HDs, or 6 sera from patients with CRC diluted at 1/1000-fold with a buffer comprising 20 mM Tris–HCl (pH 7.6), 137 mM NaCl, and 0.1% Tween 20. After incubation for 20 min with 1:30,000-diluted horseradish peroxidase-conjugated secondary antibody, immunoreactivity was detected using Immobilon Western Chemiluminescent HRP Substrate (WBKLS0500, Merck KGaA) as described previously [12, 13]. We employed BLUeye Prestained Protein Ladder (Catalog number PM007-0500, Bio-Helix, Keelung, Taiwan) as a molecular weight marker.

To examine the expression of ING1 and p53, 10 μg proteins of total cell extracts were electrophoresed, blotted, and probed with anti-ING1 (C-19, sc-7566, Santa Cruz Biotechnology, Santa Cruz, CA) and anti-p53 (DO-1, sc-126, Santa Cruz Biotechnology) antibodies. We also used anti-β-actin antibody (C11, sc-1615, Santa Cruz Biotechnology) as a loading control.

### Amplified luminescence proximity homogeneous assay-linked immunosorbent assay

We performed amplified luminescence proximity homogeneous assay-linked immunosorbent assay (AlphaLISA) using 384-well microtiter plates (white opaque OptiPlate, PerkinElmer, Waltham, MA) containing 2.5 μL 1/100-diluted sera and 2.5 μL GST-ING1 or control GST proteins (10 µg/mL), or biotinylated peptides (400 ng/mL) in AlphaLISA buffer containing 25 mM HEPES (pH 7.4), 0.1% casein, 0.5% Triton X-100, 1 mg/mL dextran-500, and 0.05% Proclin-300. We incubated the reaction mixtures at room temperature for 6 to 8 h, and then incubated them at room temperature in the dark for 7 to 21 days with anti-human IgG-conjugated acceptor beads (2.5 µL at 40 µg/mL) and glutathione-conjugated or streptavidin-conjugated donor beads (2.5 µL at 40 µg/mL). After many AlphaLISA trials, we concluded that an incubation time of 7 to 21 days was most suitable to obtain specific, stable, low background, and reproducible results. We read the Alpha photon counts in microtiter plates using an EnSpire Alpha microplate reader (PerkinElmer) as described previously [8, 9, 12–14]. Specific anti-ING1 antibody levels were obtained by subtracting the Alpha counts for the GST and buffer controls from those for the GST-ING1 protein and ING1 peptide, respectively.

### Peptide synthesis

Possible epitope sites in the ING1 protein (Accession number: NM_198219.2) were predicted using the program ProPred [15], which is a tool for predicting MHC class II binding regions in antigenic protein sequences using matrix based prediction algorithm, employing amino-acid/position coefficient table as described previously [8, 9]. The parameters are as follow; Threshold: 3%, Display top scorer: blank, Result Display Format: HTMLII, Allele: ALL. Three ING1 peptides were designed with the following amino acid sequences:

- bING1-75, biotin-QRALIRSQELGDEKI
- bING1-88, biotin-KIQIVSQMVELVENR
- bING1-239, biotin-SCVGLNHKPKGKWYC

### Cell lines and culture

We cultured human embryonic kidney 293 cells and 4 human colorectal cancer cell lines (DLD-1, Caco-2, LoVo, and HT-29) in specific culture media. 293, Caco-2, and LoVo cells were obtained from RIKEN Cell Bank (RIKEN BRC, Tsukuba, Japan). DLD-1 cells were from the American Type Culture Collection (Manassas, VA). HT-29 cells were from Sumitomo Pharma Co., Ltd. (Osaka, Japan). We cultured the 293 and DLD-1 cells in Dulbecco’s Modified Eagle’s Minimum Essential Medium (Catalog number 08456–65, Nacalal Tesque, Kyoto, Japan) supplemented with 10% fetal bovine serum (FBS) and 100 μg/ml kanamycin. We cultured Caco-2 cells in minimum essential medium (Catalog number M4655, Sigma–Aldrich, St. Louis, MO) supplemented with 20% FBS, 0.1 mM Non-Essential Amino Acids (Catalog number M7145, Sigma–Aldrich), and 100 μg/ml kanamycin. We cultured the LoVo cells in Ham’s F-12 media (Catalog number N6658, Sigma–Aldrich) supplemented with 10% FBS and 100 μg/ml kanamycin and cultured the HT-29 cells in McCoy's 5a modified medium (Catalog number 16600082, Thermo Fisher Scientific) supplemented with 10% FBS and 100 μg/ml kanamycin.

### Plasmids

The reporter plasmids, pCMV-p53WT, and pBV-PUMA-Luc [16] were provided by Dr. Bert Vogelstein (Howard Hughes Medical Institute). The plasmids pGL3-Bax-Luc and pGL3-p21-Luc [17] were provided by Dr. Mian Wu (University of Science and Technology of China), and the pGV-B2 NOXA-Luc [18] was provided by Dr. Nobuyuki Tanaka (Nippon Medical School). The plasmid pcDNA3-ING1 encoded ING1 and was expressed as described previously [19].

### Cell culture and luciferase assays

We seeded LoVo CRC cells in 24-well plates and transfected them with pcDNA3-ING1, pCMV-p53WT, both, or control empty vector plasmid (pcDNA3) with firefly luciferase and *Renilla* luciferase reporter plasmids using LipofectAMINE-Plus (Catalog number 15338100, Thermo Fisher Scientific). Two days after transfection, we measured luciferase activities using a Dual Luciferase Assay System (Catalog number E1910, Promega) and a luminescence imaging instrument (Atto, Tokyo, Japan). We normalized the firefly luciferase activities to those of the *Renilla* luciferase control as described previously [20].

### Statistical analysis

We tested the normality of each group with the Shapiro–Wilk test and the Kolmogorov–Smirnov test and selected non-parametric tests. We examined significant differences in Alpha counts with Dunn’s multiple comparison test following a Kruscal-Wallis test in comparisons between 2 groups selected from 3 or more groups. We examined simple comparisons between the 2 groups with the Mann–Whitney *U* test. We employed log-rank and generalized Wilcoxon tests to compare overall survival between the 2 groups.

## Results

### Isolation of ING1 by ProtoArray screening

ProtoArrays loaded with 9375 antigenic proteins were reacted with five each of HD and patient sera. All data of the ProtoArray are available in the public Figshare database (https://figshare.com/articles/dataset/Protein_assay_analysis_of_colorectal_cancer/21510054). We selected 4035 proteins with 0/5 positivity for HD specimens to exclude false positives. among which, we then selected 8 antigens with a 4/5 positivity rate and 135 antigens with a 3/5 positivity rate in patient specimens. No antigen with a 5/5 positivity rate was included. ING1 (Accession number: NM_198219.2), which reacted with 3/5 serum samples from patients with CRC but with 0/5 sample from HDs, is of much interest because it was related to tumor suppressor p53 [19]. GST-fusion ING1 protein was expressed in *E. coli* and then purified using affinity chromatography.

### Identification of serum s-ING1-Ab levels in patients with colorectal cancer

**Fig. 1:**
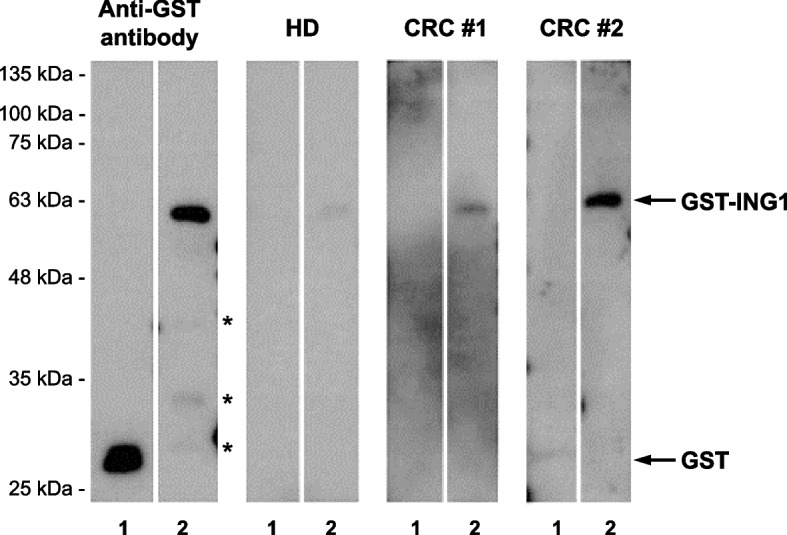
Presence of anti-ING1 antibodies in sera of patients with CRC. The reactivity of anti-ING1 antibodies against ING1 protein were examined by western blot analysis. Glutathione-S-transferase (GST) (lane 1) and GST-ING1 proteins (lane 2) were electrophoresed on sodium dodecyl sulfate–polyacrylamide gels. Anti-GST antibody (Rockland, Gilbertsville, PA), serum from healthy donors (HDs), or sera from patients with colorectal cancer (CRC) (CRC #1 and #2) were used as primary antibodies. One of the 2 independent repeat experiments is shown. Molecular sizes are shown at the left. Asterisks represent the degradation products of GST-ING1 protein. The full blot images are shown in Additional file 2: Figure S1

To determine the presence of s-ING1-Abs, we performed western blotting using sera from HDs and patients with CRC. The anti-GST antibody reacted with GST and GST-ING1 and indicated molecular weights of 28 and 65 kDa, respectively (Fig. 1). Although 65 kDa is compatible with the calculated molecular weight of GST-ING1 in the public database, preparations of GST-ING1 contained some degradation products as seen in lane 2 of the blot with anti-GST antibody shown in Fig. 1. Reactivity for 65-kDa GST-ING1 was limited in HD sera but was strong in sera from patients with CRC.

### Anti-ING1 antibody levels are increased in patients with gastrointestinal cancers

**Fig. 2:**
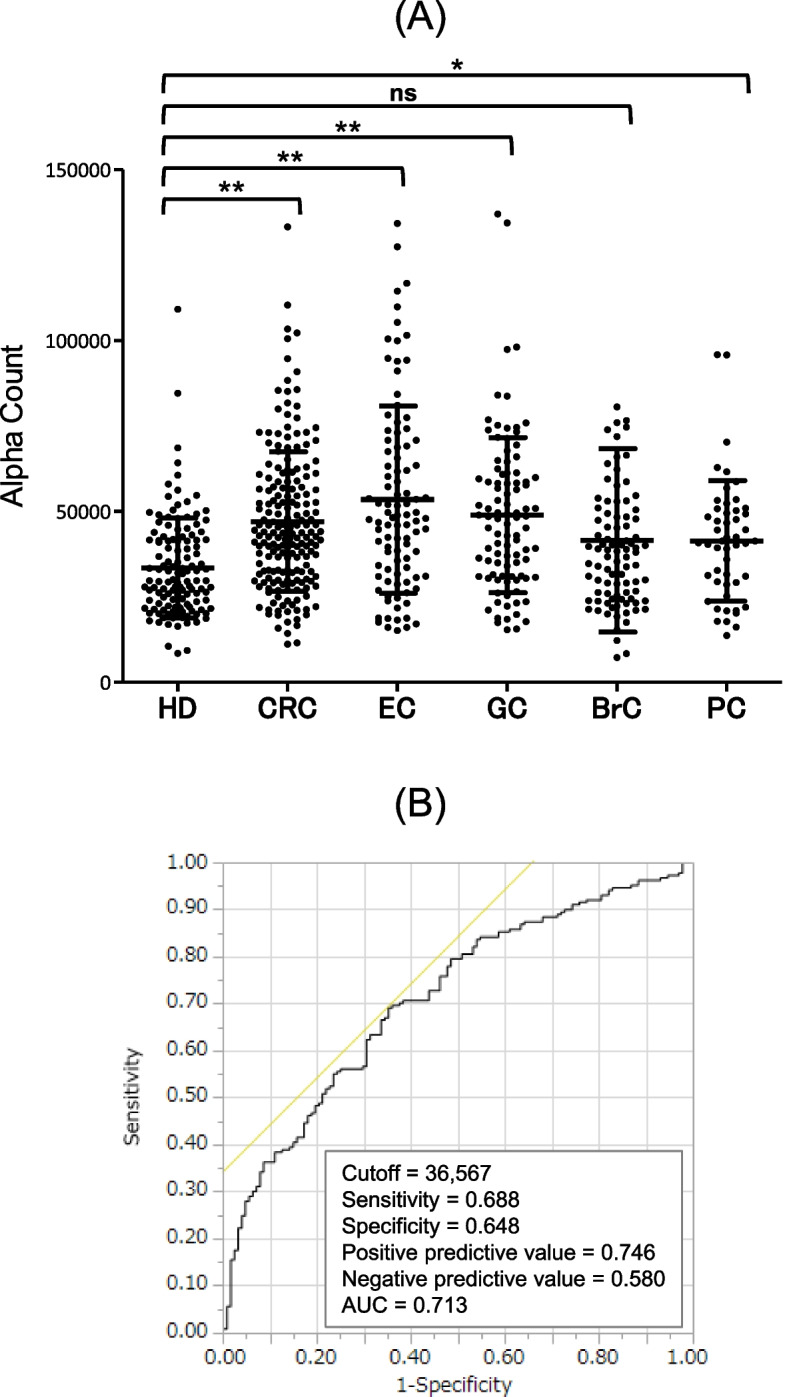
Comparison of anti-ING1 antibody levels between HDs and patients with solid cancers. **A** Serum antibody levels against ING1 protein were quantified by amplified luminescence proximity homogeneous assay-linked immunosorbent assay (AlphaLISA), and compared between HDs and patients with CRC, esophageal cancer (EC), gastric cancer (GC), breast cancer (BrC), and pancreatic cancer (PC). The Alpha photon counts represent the antibody levels, and are shown in a scatter dot plot. Horizontal lines represent the averages, and the error bars represent standard deviations (SDs). Differences were examined with Dunn’s multiple comparison test following a Kruscal-Wallis test (*p* < 0.001). *, *p* < 0.05; **, *p* < 0.001; ns, not significant. Data are summarized in Additional file 1: Table S3. **B** CRC detection using s-ING1-Abs was assessed using receiver operating characteristic (ROC) analyses. Numbers in the figures indicate cutoff values for Alpha counts, sensitivity, specificity, and area under the curve (AUC)

We determined s-ING1-Ab levels using AlphaLISA with purified recombinant GST-ING1 protein. Mean baseline characteristics of 6 cohort groups (HDs and patients with CRC, EC, GC, BrC, and PC) are shown in Additional file 1: Table S2. The s-ING1-Ab levels in the patients with CRC, EC, or GC were significantly higher than those in the HDs (Fig. 2A, Additional file 1: Table S3). Compared with the HDs, the patients with PC and BrC showed slight increases in s-ING1-Ab levels. Receiver operator characteristic (ROC) curve analysis was performed to evaluate the sensitivity and specificity for patients with CRC and HDs. The results showed the sensitivity and specificity values of 68.8% and 64.8%, respectively, using the cutoff value of 36,567 (Fig. 2B). The area under the curve value was 0.713, which suggests moderate accuracy in discriminating CRC when employing the s-ING1-Ab marker.

**Fig. 3:**
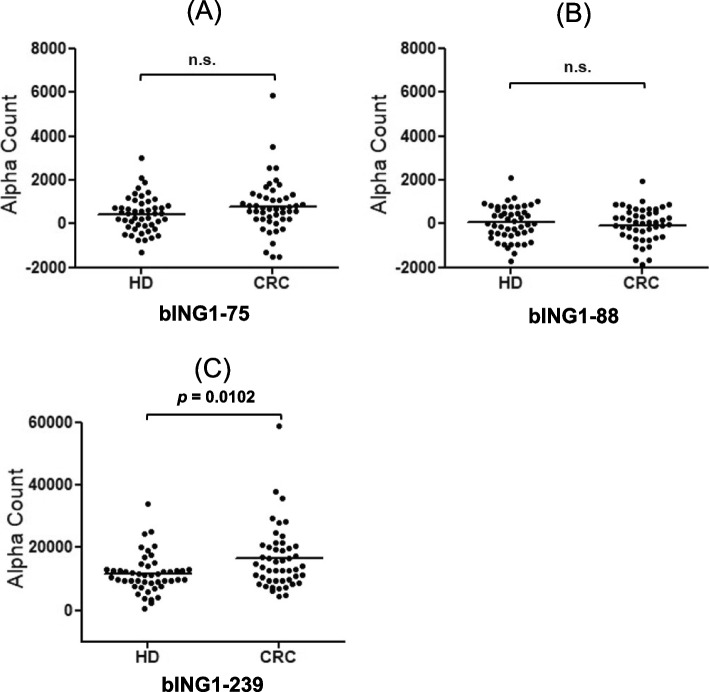
Serum antibody levels against ING1 peptides. Serum antibody levels were examined using AlphaLISA with the biotinylated peptides, bING1-75 (**A**), bING1-88 (**B**), and bING1-239 (**C**) as antigens. Preliminary experiments were performed after random selection of 48 serum samples from HDs and 48 from patients with CRC. The *p* value was calculated with the Mann–Whitney *U* test. n.s., not significant

**Fig. 4:**
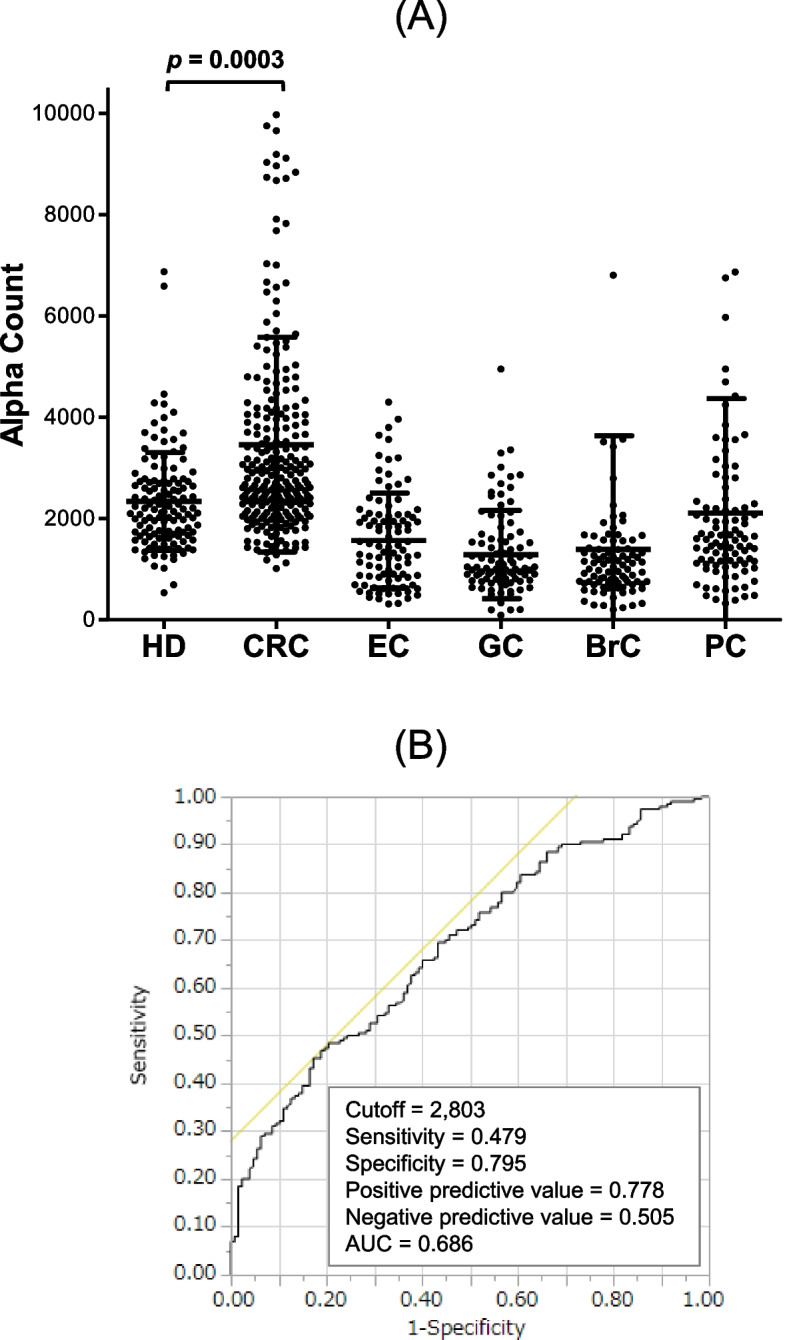
Comparison of anti-ING1-peptide antibody levels between HDs and patients with solid cancers. **A** Serum antibody levels against the bING1-239 peptide were compared between HDs and patients with solid cancers. The levels examined by AlphaLISA are shown in a scatter dot plot. Horizontal lines represent the averages, and the error bars represent SDs as described in the legends of Fig. 2. The *p* value was calculated with Dunn’s multiple comparison tests following a Kruscal-Wallis test (*p* < 0.001). Data are summarized in Additional file 1: Table S3. **B** CRC detection using anti-bING1 peptide antibodies was assessed using ROC analysis. Numbers in the figures indicate cutoff values, sensitivity, specificity, and AUC value

To improve the specificity of reactions, we identified 3 candidate ING1 epitopes using the program ProPred and synthesized the corresponding biotinylated peptides. Subsequently, we examined antibody levels against these peptides in 48 serum samples from HDs and another 48 from patients with CRC using AlphaLISA. We observed a significant difference in the antibody levels between HDs and patients against the bING1-239 peptide but not against bING1-75 or bING1-88 (Fig. 3A–C). We then measured serum antibody levels in all cancer samples with the bING1-239 peptide. The results showed that anti-bING1-239 antibody levels were significantly higher only in patients with CRC but not in those with other cancers, including EC, GC, BrC, and PC or in HDs (Fig. 4A, Additional file 1: Table S3). ROC analyses of anti-bING1-239 antibodies versus CRC showed sensitivity and specificity values of 47.9% and 79.5%, respectively (Fig. 4B).

**Fig. 5:**
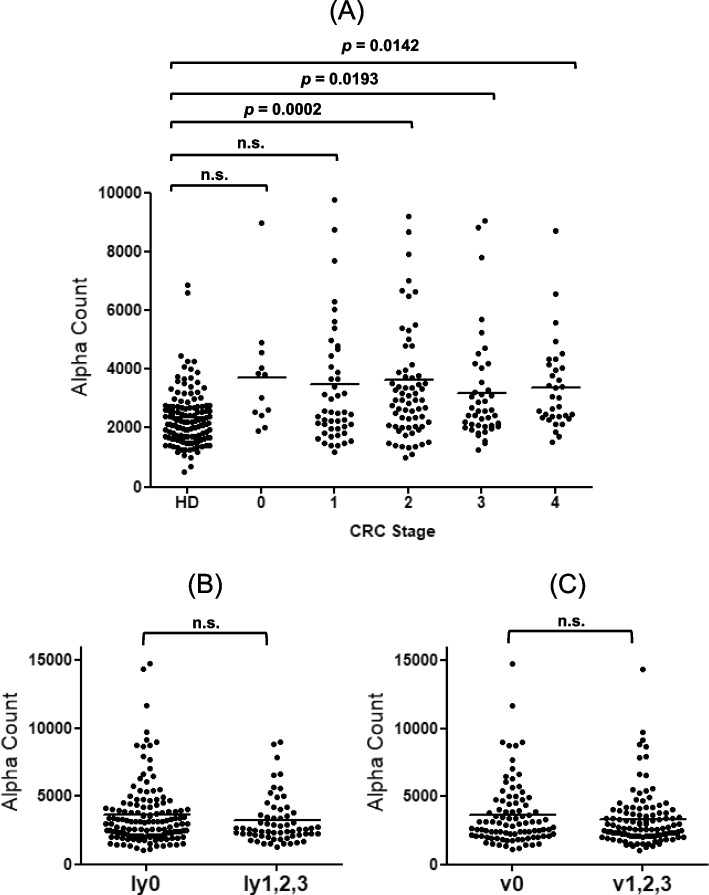
Comparison of s-ING1-Ab levels among stages of CRC. Serum antibody levels against the bING1-239 peptide were compared among pathological properties including **A** pathological stages, **B** lymphatic invasion (ly), and **C** venous invasion (v) in 133 patients with CRC. Patients treated with chemotherapy and/or palliative colostomy only or with neo adjuvant therapy were excluded. The sample number of each stage and invasion status are shown in Additional file 1: Table S4. Comparisons were made using Dunn’s multiple comparison test following a Kruscal-Wallis test (*p* < 0.001). (**A**), and the Mann–Whitney *U* test (**B**, **C**)

**Fig. 6:**
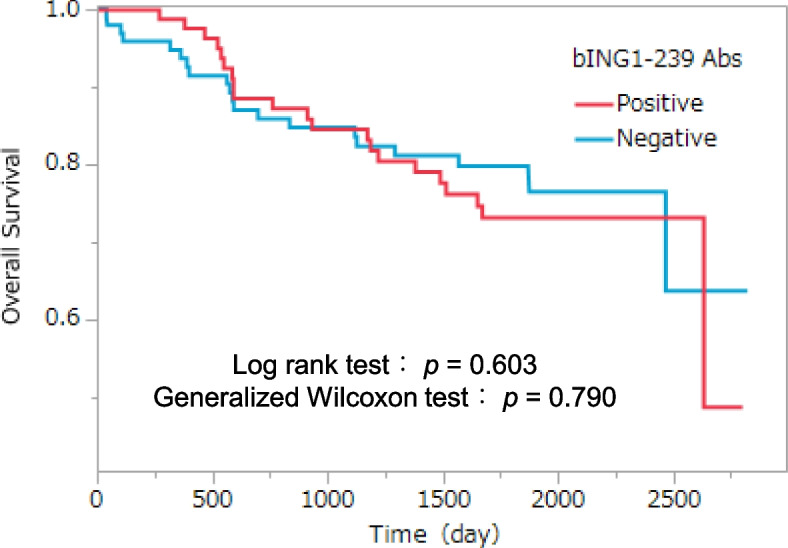
Kaplan–Meier overall survival curves for patients with or without serum bING1-239 antibodies. The cutoff value was determined by ROC analysis. The *p* value was calculated using log-rank and generalized Wilcoxon tests

We then identified relationships between the presence of s-ING1-Abs and pathological stages and lymphatic and venous invasions. Patients with any stagesof CRC showed significantly higher levels of s-ING1-Abs than HDs (Fig. 5A), indicating that s-ING1-Ab levels can be used to identify the presence of CRC at as early stage. No significant differences were observed among the various CRC stages. Lymphatic and venous invasions were not apparently correlated with s-ING1-Ab levels (Fig. 5B and C), and Kaplan–Meier plots showed no significant correlation between s-ING1-Ab levels and survival (Fig. 6).

### Expression of ING1 in colorectal cancer tissues and colorectal cancer cell lines

**Fig. 7:**
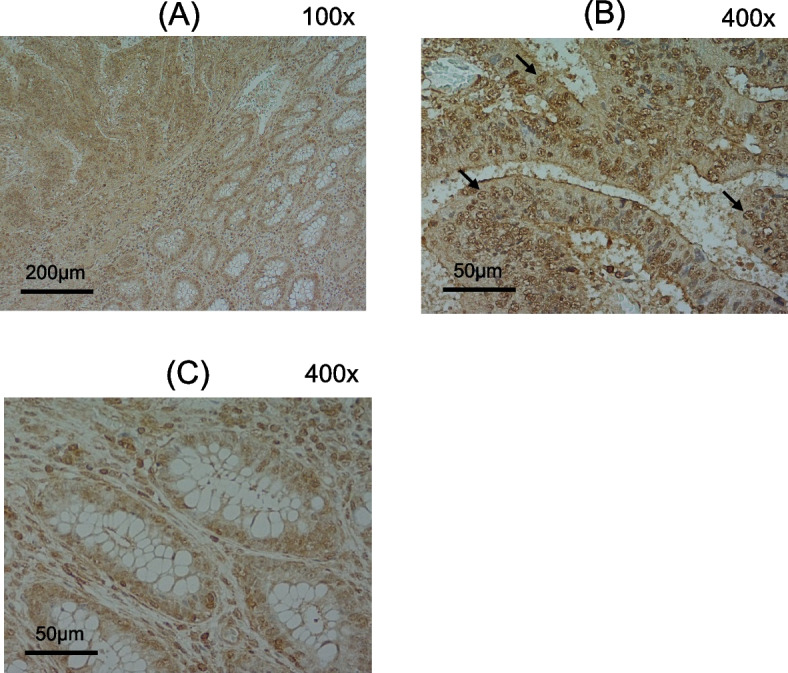
Immunohistochemical staining of normal and cancer tissues from patients with colorectal cancer. Immunohistochemical staining was performed on 6 resection specimens randomly selected from 192 patients with CRC. One representative result is shown. **A** cancer (upper left) and normal (lower right) tissue; **B** cancer tissue; **C** normal tissue. Some of typical localizations in nuclei are indicated by arrows in (**B**)

We examined expression levels and distributions of ING1 proteins in CRC specimens using immunohistochemical staining with an anti-human ING1 mouse monoclonal antibody (R&D Systems). In these analyses of formalin-fixed tissue specimens containing cancerous and normal cells from patients with CRC, more intense staining was shown in CRC tissues (Fig. 7A, upper left part) than in normal colorectal tissues (Fig. 7A, lower right part), as shown in representative tissue sections. ING1 proteins were localized predominantly in the nuclei of CRC and normal colorectal cells (Fig. 7B and C).

**Fig. 8:**
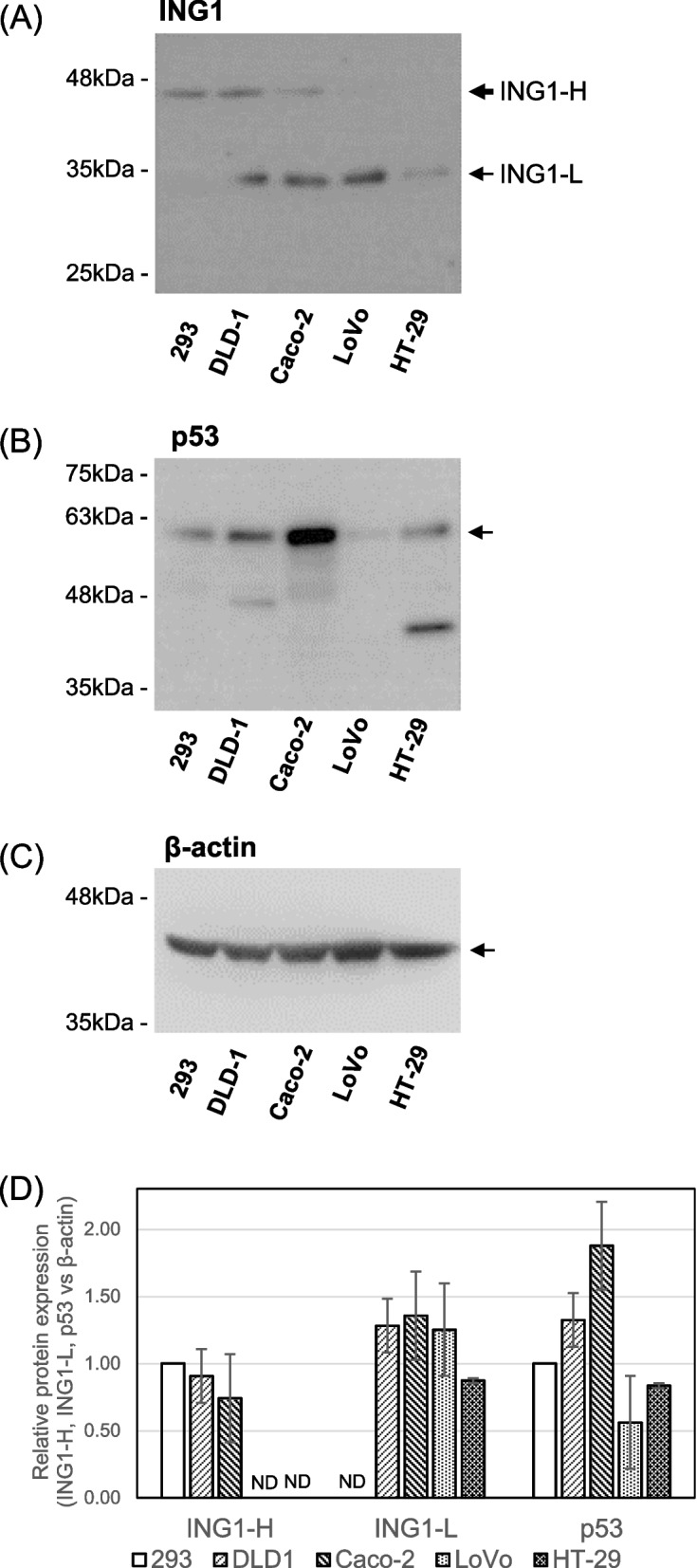
The expression of ING1 and p53 proteins in CRC cell lines. The expression levels of ING1 and p53 protein as well as a loading control, β-actin, in human embryonic kidney, 293 cells, and CRC cell lines (DLD-1, Caco-2, LoVo, and HT-29) (10 μg protein of total cell extracts) were examined by western blotting using anti-human ING1 (C19, sc-7566, Santa Cruz Biotechnology) (**A**), anti-human p53 (DO-1, sc-126, Santa Cruz Biotechnology) (**B**), and anti-β-actin (C11, sc-1615, Santa Cruz Biotechnology) antibodies (**C**). The positions of p47-ING1 (ING1-H), p33-ING1 (ING1-L), p53, and β-actin proteins are indicated by arrows. **D** Quantification of the results for ING1-H, ING1-L, and p53 versus β-actin. ING1-H/β-actin and p53/β-actin were normalized to 1.0. Values are expressed as the average ± SD of 3 independent experiments. ND, not detectable

To examine the relationship between ING1 and p53 protein in CRC, we determined expression levels in 4 CRC cell lines and in 293 cells using western blotting. The p33ING1 and p47ING1 isoforms were expressed in these lines and p33ING1 was observed in all CRC cell lines but not in immortalized HEK293 cells (Fig. 8A). Similarly, p53 was highly expressed in the 293, DLD-1, CACO-2, and HT-29 CRC cell lines, whereas comparatively low expression levels of p53 were observed in the LoVo cells (Fig. 8B, C and D). These data are consistent with a previous report that LoVo cells but not other CRC cells carry the wild-type *p53* gene [21, 22]. The expression levels of ING1 isoforms were independent of *p53* mutations, which is compatible with the report of Hara et al. [23].

### Functional analysis of ING1 using reporter assays

**Fig. 9:**
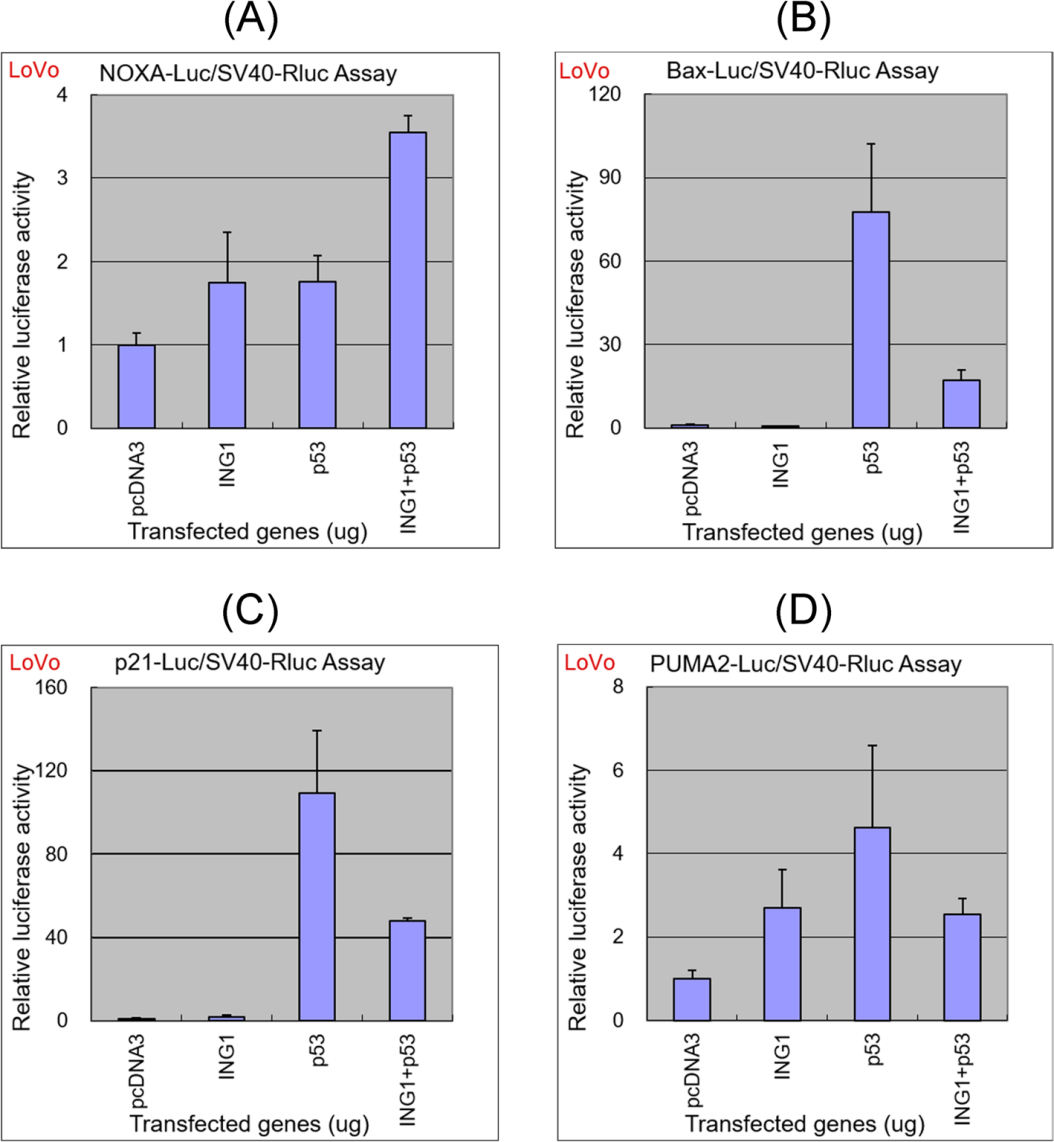
Luciferase assays. LoVo CRC cells were seeded and transfected with the luciferase reporter plasmids NOXA-Luc (**A**), Bax-Luc (**B**), p21-Luc (**C**), and PUMA2-Luc (**D**) with or without the expression plasmids of ING1 and/or p53. Cells were harvested 48 h after transfection and luciferase activities in the cell lysates were measured. Relative luciferase activities in cells transfected with ING1 and/or p53 versus those in cells transfected with empty vector pcDNA3 are shown. Each column and bar represent the average and SD, respectively, of 3 independent experiments

We transfected LoVo cells with a control vector or vectors encoding ING1 or p53 or both, and quantified transactivation of p53 response elements using luciferase reporters. NOXA-Luc, Bax-Luc, p21-Luc, and PUMA-Luc promoters are typical reporters for p53 and were highly activated following transfection with p53 construct. Moreover, enforced expression of p53 activated the NOXA-Luc promoter, which was further activated by co-transfection with ING1 (Fig. 9A), suggesting that p53 was activated by ING1, as reported previously [19]. Luciferase activities of Bax-Luc, p21-Luc, and PUMA2-Luc were also elevated by transfection with p53 alone, but the levels were attenuated by co-transfection with ING1 (Fig. 9B–D).

## Discussion

In this study, we performed ProtoArray screening analyses and identified autoantibodies against p33ING1 in sera from patients with CRC as a candidate marker. Therefore, we targeted p33ING1 for subsequent experiments, but other ING1 variants, p24 and p47, may also affect p33 function. The role of other variants as well as their antibodies should also be investigated in future work. Another point to consider is that the number of specimens in the first screening was small and there may be other candidate markers besides s-ING1-Ab. Other antibody marker candidates should be explored in the future by conducting ProtoArray screening analyses using more serum samples. A subsequent AlphaLISA showed that s-ING1-Ab levels against ING1 were markedly elevated in sera from patients with CRC compared with those in serum from HDs (Fig. 2). Furthermore, experiments using the bING1-239 peptide showed that s-ING1-Ab levels were significantly elevated in sera from patients with CRC, whereas these levels were not significantly higher in sera from patients with other cancers (EC, GC, BrC, and PC) than in those from HDs (Fig. 4A, Additional file 1: Table S3). Therefore, s-ING1-Abs have potential as a novel and specific biomarker for CRC. Because ING1 protein between amino acid 239 and 253 is highly conserved among members of the ING family (ING1-5) [24], the antibody levels examined by using ING1 protein and bING1-239 peptide as substrates may involve antibodies against other ING family members but not be specific for ING1 antibodies. The relationship between s-ING1-Ab levels and the expression of ING family members other than ING1 should be investigated in future studies. Although the sensitivity and specificity of s-ING1-Abs for CRC were not compelling, s-ING1-Ab levels > 6000 were almost exclusively observed in sera from patients with CRC (Fig. 4), suggesting that the major part, if not all, of s-ING1-Ab highly positive subjects are sera from CRC patients. Although ING1 has also been identified as a tumor antigen in BrC [25], s-ING1-Ab levels in patients with BrC were not high (Figs. 2A and 4A). Further study is necessary to clarify the relationship of the levels between ING1 protein expression and s-ING1-Abs in CRC and BrC.

Current tumor diagnosis methods employ various antigen markers; however, antibody markers have not been put into practical use except for anti-p53 antibodies [3, 26]. Repeated tissue destruction of cancer cells even at the early-stage induces repeated leakage of small amounts of otherwise intracellular antigenic proteins, which increases the antibodies to the detectable levels. The s-ING1-Ab levels were consistently and significantly increased in sera from patients with CRC at any stages compared with HDs (Fig. 5A). Thus, the s-ING1-Ab marker can detect CRC as early stage. However, the sample size for stage 0 of CRC is 12 cases, which is fewer than the other stages. Therefore, additional experiments with more samples should be conducted in the future. In our clinical analyses, s-ING1-Ab levels did not increase with CRC progression, and Kaplan–Meier plots showed no significant differences in survival between positive and negative patients (Fig. 6). Early detection of CRC using the s-ING1-Ab marker could possibly improve prognoses by signaling the need for treatment at earlier stages of malignancy. The cause of the development of s-ING1-Abs remains to be explained. Simple tissue destruction may not be sufficient to increase antibody levels, and it is possible that destruction of cells with high ING1 expression results in increased antibody levels. This inference should be explored in future experiments.

ING1 is known as a tumor suppressor [27, 28] and is mainly localized in the nucleus via a nuclear localization signal in the middle part [27], as observed in Fig. 7A–C. ING1 is involved in the regulation of proliferation, cell cycle, senescence, apoptosis, chromatin remodeling, DNA repair, and genome stability [28, 29]. Functional cooperation of p53 and ING1 has been associated with apoptosis induction and growth inhibition, and physical interactions between these proteins have been reported [30, 31]. Moreover, enforced expression of ING1 has been shown to activate the p53 target gene in multiple studies [32, 33]. However, induction of p53-independent apoptosis by ING1 has also been demonstrated, and translocation of ING1 into mitochondria was followed by increased Bax levels in mitochondrial membranes and consequent induction of apoptosis [30, 33–35]. In our luciferase reporter assays, the promoter activity of the pro-apoptotic NOXA was increased additively by ING1 and p53 (Fig. 9A). These data suggest that ING1 activates p53, although it cannot be ruled out the possibility that ING1 and p53 activated NOXA promoter via different pathways. In contrast, the activation of the promoters of pro-apoptotic Bax and PUMA and growth-inhibitory p21 by p53 was attenuated by co-transfection with ING1-encoding vectors (Fig. 9B–D). Thus, ING1 does not necessarily induce apoptosis but may inhibit cell growth in wild-type p53 harboring cells. This should be further investigated in the future.

We have reported many EC antibody biomarkers identified by SEREX screening, most of which were more or less responsive to other digestive-organ cancers such as GC and CRC [36]. Herein, we show that serum levels of antibodies against the bING1-239 peptide were specifically elevated in sera from patients with CRC but not elevated in sera from patients with EC, GC, BrC, or PC (Fig. 4A). Moreover, elevated s-ING1-Ab levels were associated with any CRC stages (Fig. 5A). Therefore, the present s-ING1-Ab marker could be especially useful for specific and early diagnosis of CRC.

## Supplementary Information

**Additional file 1:** **Table S1.** List of antibodies. **Table S2.** Baseline characteristics of subjects. Numbers, ages (average ± SD), and sexes of patients in each group are presented. No significant Spearman’s correlation was identified between s-ING1-Abs levels and sexes or ages of patients. **Table S3.** Comparison of s-ING1-Ab levels between HD and patients with CRC or other cancers were examined using AlphaLISA. Data are those shown in Figs. 2 and 4. Averages, SDs, cutoff values, total sample numbers, positive numbers, positive rates, and p values are presented. Four samples (one from an HD, two from CRC patients, one from an EC patient) were excluded as outliers owing to high values of the Buffer control that exceeded 10 SD of the Alpha count in serum from HDs. The cutoff value in this table was determined as the mean + 2 SD of Alpha counts in serum from HD. **Table S4.** Pathological properties of 133 CRC. The number of each stage, lymphatic invasion, and venous invasion. n.d., no data.

**Additional file 2:** **Figure S1.** Full blot images of Fig. 1. The reactivity of anti-ING1 antibodies against ING1 protein were examined by western blot analysis. GST (lane1) and GST-ING1 proteins (lane 2) were electrophoresed on sodium dodecylsulfate-polyacrylamide gels. Anti-GST antibody, sera from healthy donors (HD #1 and #2), or sera from patients with colorectalcancer (CRC) (CRC #1–#5) were used as primary antibodies. Molecular sizes are shown at the left.

## Data Availability

All data of the ProtoArray v5.1 human protein microarray system are available in the public Figshare database (https://figshare.com/articles/dataset/Protein_assay_analysis_of_colorectal_cancer/21510054).

## References

[1] Ferlay J, Colombet M, Soerjomataram I, Mathers C, Parkin DM, Piñeros M, Znaor A, Bray F (2018). Estimating the global cancer incidence and mortality in 2018: GLOBOCAN sources and methods. Int J Cancer.

[2] Mandel JS, Bond JH, Church TR, Snover DC, Bradley GM, Schuman LM, Ederer F (1993). Reducing Mortality from colorectal cancer by screening for fecal occult blood. Minnesota Colon Cancer Control Study. N Engl J Med..

[3] Zamcheck N, Pusztaszeri G (1975). CEA, AFP and other potential tumor markers. CA Cancer J Clin.

[4] Kuusela P, Jalanko H, Roberts P, Sipponen P, Mecklin JP, Pitkänen R, Mäkelä O (1984). Comparison of CA 19–9 and carcinoembryonic antigen (CEA) levels in the serum of patients with colorectal diseases. Br J Cancer.

[5] Shimada H, Takeda A, Arima M, Okazumi S, Matsubara H, Nabeya Y, Funami Y, Hayashi H, Gunji Y, Suzuki T (2000). Serum p53 antibody is a useful tumor marker in superficial esophageal squamous cell carcinoma. Cancer.

[6] Takeda A, Koyama I, Shimada H, Ochiai T (2007). Titration of serum p53 autoantibodies in patients with colorectal cancer and the clinical significance of post-operative monitoring. Nippon Daicho Komonbyo Gakkai Zasshi.

[7] Kagaya A, Shimada H, Shiratori T, Kuboshima M, Nakashima-Fujita K, Yasuraoka M, Nishimori T, Kurei S, Hachiya T, Murakami A (2011). Identification of a novel SEREX antigen family, ECSA, in esophageal squamous cell carcinoma. Proteome Sci.

[8] Naito A, Hiwasa T, Tanabe N, Sanada TJ, Sugiura T, Shigeta A, Terada J, Takizawa H, Kashiwado K, Sakao S (2019). Elevated levels of autoantibodies against EXD2 and PHAX in the sera of patients with chronic thromboembolic pulmonary hypertension. PLoS ONE.

[9] Hiwasa T, Wang H, Goto K, Mine S, Machida T, Kobayashi E, Yoshida Y, Adachi A, Matsutani T, Sata M (2021). Serum anti-DIDO1, anti-CPSF2, and anti-FOXJ2 antibodies as predictive risk markers for acute ischemic stroke. BMC Med.

[10] Shimada H, Shiratori T, Yasuraoka M, Kagaya A, Kuboshima M, Nomura F, Takiguchi M, Ochiai T, Matsubara H, Hiwasa T (2009). Identification of makorin 1 as a novel SEREX antigen of esophageal squamous cell carcinoma. BMC Cancer..

[11] Machida T, Kubota M, Kobayashi E, Iwadate Y, Saeki N, Yamaura A, Nomura F, Takiguchi M, Hiwasa T (2015). Identification of stroke-associated-antigens via screening of recombinant proteins from the human expression cDNA library (SEREX). J Transl Med.

[12] Wang H, Zhang XM, Tomiyoshi G, Nakamura R, Shinmen N, Kuroda H, Kimura R, Mine S, Kamitsukasa I, Wada T (2018). Association of serum levels of antibodies against MMP1, CBX1, and CBX5 with transient ischemic attack and cerebral infarction. Oncotarget.

[13] Yoshida Y, Zhang XM, Wang H, Machida T, Mine S, Kobayashi E, Adachi A, Matsutani T, Kamitsukasa I, Wada T (2020). Elevated levels of autoantibodies against DNAJC2 in sera of patients with atherosclerotic diseases. Heliyon.

[14] Li SY, Yoshida Y, Kobayashi E, Kubota M, Matsutani T, Mine S, Machida T, Maezawa Y, Takemoto M, Yokote K (2021). Serum anti-AP3D1 antibodies are risk factors for acute ischemic stroke related with atherosclerosis. Sci Rep.

[15] Singh H, Raghava GP (2001). ProPred: prediction of HLA-DR binding sites. Bioinformatics.

[16] Nakano K, Vousden KH (2001). PUMA, a novel proapoptotic gene, is induced by p53. Mol Cell.

[17] Jiang P, Du W, Heese K, Wu M (2006). The Bad guy cooperates with good cop p53: Bad is transcriptionally up-regulated by p53 and forms a Bad/p53 complex at the mitochondria to induce apoptosis. Mol Cell Biol.

[18] Oda E, Ohki R, Murasawa H, Nemoto J, Shibue T, Yamashita T, Tokino T, Taniguchi T, Tanaka N (2000). Noxa, a BH3-only member of the Bcl-2 family and candidate mediator of p53-induced apoptosis. Science.

[19] Shimada H, Liu TL, Ochiai T, Shimizu T, Haupt Y, Hamada H, Abe T, Oka M, Takiguchi M, Hiwasa T (2002). Facilitation of adenoviral wild-type p53-induced apoptotic cell death by overexpression of p33^ING1^ in T.Tn human esophageal carcinoma cells. Oncogene..

[20] Shinmen N, Koshida T, Kumazawa T, Sato K, Shimada H, Matsutani T, Iwadate Y, Takiguchi M, Hiwasa T (2009). Activation of NFAT signal by p53–K120R mutant. FEBS Lett.

[21] Ahmed D, Eide PW, Eilertsen IA, Danielsen SA, Eknæs M, Hektoen M, Lind GE, Lothe RA (2013). Epigenetic and genetic features of 24 colon cancer cell lines. Oncogenesis.

[22] Nita ME, Nagawa H, Tominaga O, Tsuno N, Fujii S, Sasaki S, Fu CG, Takenoue T, Tsuruo T, Muto T (1998). 5-Fluorouracil induces apoptosis in human colon cancer cell lines with modulation of Bcl-2 family proteins. Br J Cancer.

[23] Hara Y, Zheng Z, Evans SC, Malatjalian D, Riddell DC, Guernsey DL, Wang LD, Riabowol K, Casson AG (2003). ING1 and p53 tumor suppressor gene alterations in adenocarcinomas of the esophagogastric junction. Cancer Lett.

[24] He GH, Helbing CC, Wagner MJ, Sensen CW, Riabowol K (2005). Phylogenetic analysis of the ING family of PHD finger proteins. Mol Biol Evol.

[25] Jäger D, Stockert E, Scanlan MJ, Güre AO, Jäger E, Knuth A, Old LJ, Chen YT (1999). Cancer-testis antigens and ING1 tumor suppressor gene product are breast cancer antigens: characterization of tissue-specific ING1 transcripts and a homologue gene. Cancer Res.

[26] Shimada H, Yajima S, Oshima Y, Hiwasa T, Tagawa M, Matsushita K, Nomura F (2012). Impact of serum biomarkers on esophageal squamous cell carcinoma. Esophagus.

[27] Garkavtsev I, Kazarov A, Gudkov A, Riabowol K (1996). Suppression of the novel growth inhibitor p33^ING1^ promotes neoplastic transformation. Nat Genet.

[28] Guérillon C, Larrieu D, Pedeux R (2013). ING1 and ING2: multifaceted tumor suppressor genes. Cell Mol Life Sci.

[29] Jacquet K, Binda O (2021). ING proteins: Tumour suppressors or oncoproteins. Cancers (Basel).

[30] Bose P, Thakur S, Thalappilly S, Ahn BY, Satpathy S, Feng X, Suzuki K, Kim SW, Riabowol K (2013). ING1 induces apoptosis through direct effects at the mitochondria. Cell Death Dis.

[31] Garkavtsev I, Grigorian IA, Ossovskaya VS, Chernov MV, Chumakov PM, Gudkov AV (1998). The candidate tumour suppressor p33^*ING1*^ cooperates with p53 in cell growth control. Nature.

[32] Cheung KJ, Li G (2002). p33^*ING1*^ enhances UVB-induced apoptosis in melanoma cells. Exp Cell Res.

[33] Kataoka H, Bonnefin P, Vieyra D, Feng X, Hara Y, Miura Y, Joh T, Nakabayashi H, Vaziri H, Harris CC, Riabowol K (2003). ING1 represses transcription by direct DNA binding and through effects on p53. Cancer Res.

[34] Helbing CC, Veillette C, Riabowol K, Johnston RN, Garkavtsev I (1997). A novel candidate tumor suppressor, *ING1*, is involved in the regulation of apoptosis. Cancer Res.

[35] Coles AH, Liang H, Zhu Z, Marfella CGA, Kang J, Imbalzano AN, Jones SN (2007). Deletion of p37 ^Ing1^ in mice reveals a p53-independent role for Ing1 in the suppression of cell proliferation, apoptosis, and tumorigenesis. Cancer Res.

[36] Kobayashi S, Hiwasa T, Arasawa T, Kagaya A, Ishii S, Shimada H, Ito M, Suzuki M, Kano M, Rahmutulla B (2018). Identification of specific and common diagnostic antibody markers for gastrointestinal cancers by SEREX screening using testis cDNA phage library. Oncotarget.

