# Supplementary material for: Analysis of patients with colorectal cancer shows a specific increase in serum anti-ING1 autoantibody levels
Source: BMC Cancer. 2023 Apr 18;23:356. doi: 10.1186/s12885-023-10845-y (PMC10111810; doi:10.1186/s12885-023-10845-y)
Supplement: Supplementary file 1 — Additional file 1: Table S1. List of antibodies. Table S2. Baseline characteristics of subjects. Numbers, ages (average ± SD), and sexes of patients in each group are presented. No significant Spearman’s correlation was identified between s-ING1-Abs levels and sexes or ages of patients. Table S3. Comparison of s-ING1-Ab levels between HD and patients with CRC or other cancers were examined using AlphaLISA. Data are those shown in Figs. 2 and 4. Averages, SDs, cutoff values, total sample numbers, positive numbers, positive rates, and p values are presented. Four samples (one from an HD, two from CRC patients, one from an EC patient) were excluded as outliers owing to high values of the Buffer control that exceeded 10 SD of the Alpha count in serum from HDs. The cutoff value in this table was determined as the mean + 2 SD of Alpha counts in serum from HD. Table S4. Pathological properties of 133 CRC. The number of each stage, lymphatic invasion, and venous invasion. n.d., no data. [file 12885_2023_10845_MOESM1_ESM.docx]

**Additional file 1: Table S1.** List of antibodies.

|  | Antigen | Host | Catalog number | Maker | City/state/country |
| --- | --- | --- | --- | --- | --- |
| Primary antibody for Immunostaining | ING1 | mouse | 585922 | R&D Systems | Minneapolis/MN/USA |
| Primary antibody for Western blot | GST | goat | 600-101-200 | Rockland | Gilbertsville/PA/USA |
|  | ING1 | goat | C-19, sc-7566 | Santa Cruz Biotechnology | Santa Cruz/CA/USA |
|  | p53 | mouse | DO-1, sc-126 | Santa Cruz Biotechnology | Santa Cruz/CA/USA |
|  | β-actin | rabbit | C11, sc-1615 | Santa Cruz Biotechnology | Santa Cruz/CA/USA |
| Secondary antibody for Western blot | rabbit IgG | goat | sc-2054 | Santa Cruz Biotechnology | Santa Cruz/CA/USA |
|  | mouse IgG | goat | sc-2055 | Santa Cruz Biotechnology | Santa Cruz/CA/USA |
|  | goat IgG | donkey | sc-2056 | Santa Cruz Biotechnology | Santa Cruz/CA/USA |

**Additional file 1: Table S2.** Baseline characteristics of subjects. Numbers, ages (average ± SD), and sexes of patients in each group are presented. No significant Spearman’s correlation was identified between s-ING1-Abs levels and sexes or ages of patients.

|  | HD  (n = 128) | CRC  (192) | EC  (96) | GC  (96) | BrC  (93) | PC  (50) |
| --- | --- | --- | --- | --- | --- | --- |
| Age　mean | 58 | 66 | 67 | 70 | 56 | 68 |
| (±SD) | (±5.7) | (±12.3) | (±8.9) | (±10.2) | (±13.7) | (±10.5) |
| ≥65 (%) | 18 (14.1) | 124(64.6) | 67 (69.8) | 71 (74.0) | 29 (31.2) | 35 (70.0) |
| Sex　Men (%) | 73 (57.0) | 110(57.3) | 85 (88.5) | 65 (67.7) | 0 (0) | 30 (60.0) |

**Additional file 1: Table S3.** Comparison of s-ING1-Ab levels between HD and patients with CRC or other cancers were examined using AlphaLISA. Data are those shown in Figs. 2 and 4. Averages, SDs, cutoff values, total sample numbers, positive numbers, positive rates, and p values are presented. Sensitivity, specificity, positive predictive value, and negative predictive value in CRC patients are also presented. Four samples (one from a HD, two from CRC patients, one from an EC patient) were excluded as outliers owing to high values of the Buffer control that exceeded 10 SD of the Alpha count in serum from HDs. The cutoff value in this table was determined as the mean + 2 SD of Alpha counts in sera from HDs.

| Alpha analysis (antibody level) | | anti-ING1 protein | anti-bING1-239 |
| --- | --- | --- | --- |
| HD | Average | 33,478 | 2,337 |
|  | SD | 14,611 | 964 |
|  | Cutoff value | 62,701 | 4,265 |
|  | Positive / Total number | 4 / 128 | 4 / 127 |
|  | Positive rate (%) | 3.1 | 3.1 |
| CRC | Average | 47,885 | 3,431 |
|  | SD | 20,767 | 2,151 |
|  | Positive / Total number | 37 / 192 | 52 / 190 |
|  | Sensitivity (Positive rate) (%) | 19.3 | 27.4 |
|  | Specificity (%) | 96.9 | 96.9 |
|  | Positive predictive value (%) | 90.2 | 92.9 |
|  | Negative predictive value (%) | 44.1 | 47.1 |
| EC | Average | 53,465 | 1,564 |
|  | SD | 27,398 | 937 |
|  | Positive / Total number | 29 / 96 | 1 / 95 |
|  | Positive rate (%) | 30.2 | 1.0 |
| GC | Average | 48,959 | 1,288 |
|  | SD | 22,695 | 870 |
|  | Positive / Total number | 20 / 96 | 1 / 96 |
|  | Positive rate (%) | 20.8 | 1.0 |
| BrC | Average | 42,370 | 1,372 |
|  | SD | 26,882 | 2,269 |
|  | Positive / Total number | 11 / 93 | 3 / 93 |
|  | Positive rate (%) | 13.5 | 3.2 |
| PC | Average | 45,678 | 2,173 |
|  | SD | 23,083 | 2,876 |
|  | Positive / Total number | 16 / 50 | 4 / 50 |
|  | Positive rate (%) | 16.7 | 8.0 |

**Additional file 1: Table S4.** Pathological properties of 133 CRC. The number of each stage, lymphatic invasion, and venous invasion. n.d., no data.

|  | Lymphatic invasion | Venous invasion | Total  number |
| --- | --- | --- | --- |
| Pathological stage | ( Positive / Negative / n.d.) | ( Positive / Negative / n.d. ) |  |
| 0 | ( 0 / 8 / 0 ) | ( 0 / 8 / 0 ) | 8 |
| 1 | ( 4 / 28 / 0 ) | ( 14 / 17 / 1 ) | 32 |
| 2 | ( 7 / 35 / 0 ) | ( 19 / 23 / 0 ) | 42 |
| 3 | ( 14 / 15 / 1 ) | ( 24 / 6 / 0 ) | 30 |
| 4 | ( 16 / 5 / 0 ) | ( 19 / 2 / 0 ) | 21 |
| Total number | ( 41 / 91 / 1 ) | ( 76 / 56 / 1 ) | 133 |
