# Supplementary material for: Analysis of patients with colorectal cancer shows a specific increase in serum anti-ING1 autoantibody levels
Source: BMC Cancer. 2023 Apr 18;23:356. doi: 10.1186/s12885-023-10845-y (PMC10111810; doi:10.1186/s12885-023-10845-y)
Supplement: Supplementary file 2 — Additional file 2: Figure S1. Full blot images of Fig. 1. The reactivity of anti-ING1 antibodies against ING1 protein were examined by western blot analysis. GST (lane1) and GST-ING1 proteins (lane 2) were electrophoresed on sodium dodecylsulfate-polyacrylamide gels. Anti-GST antibody, sera from healthy donors (HD #1 and #2), or sera from patients with colorectalcancer (CRC) (CRC #1–#5) were used as primary antibodies. Molecular sizes are shown at the left. [file 12885_2023_10845_MOESM2_ESM.pptx]

## Slide 1
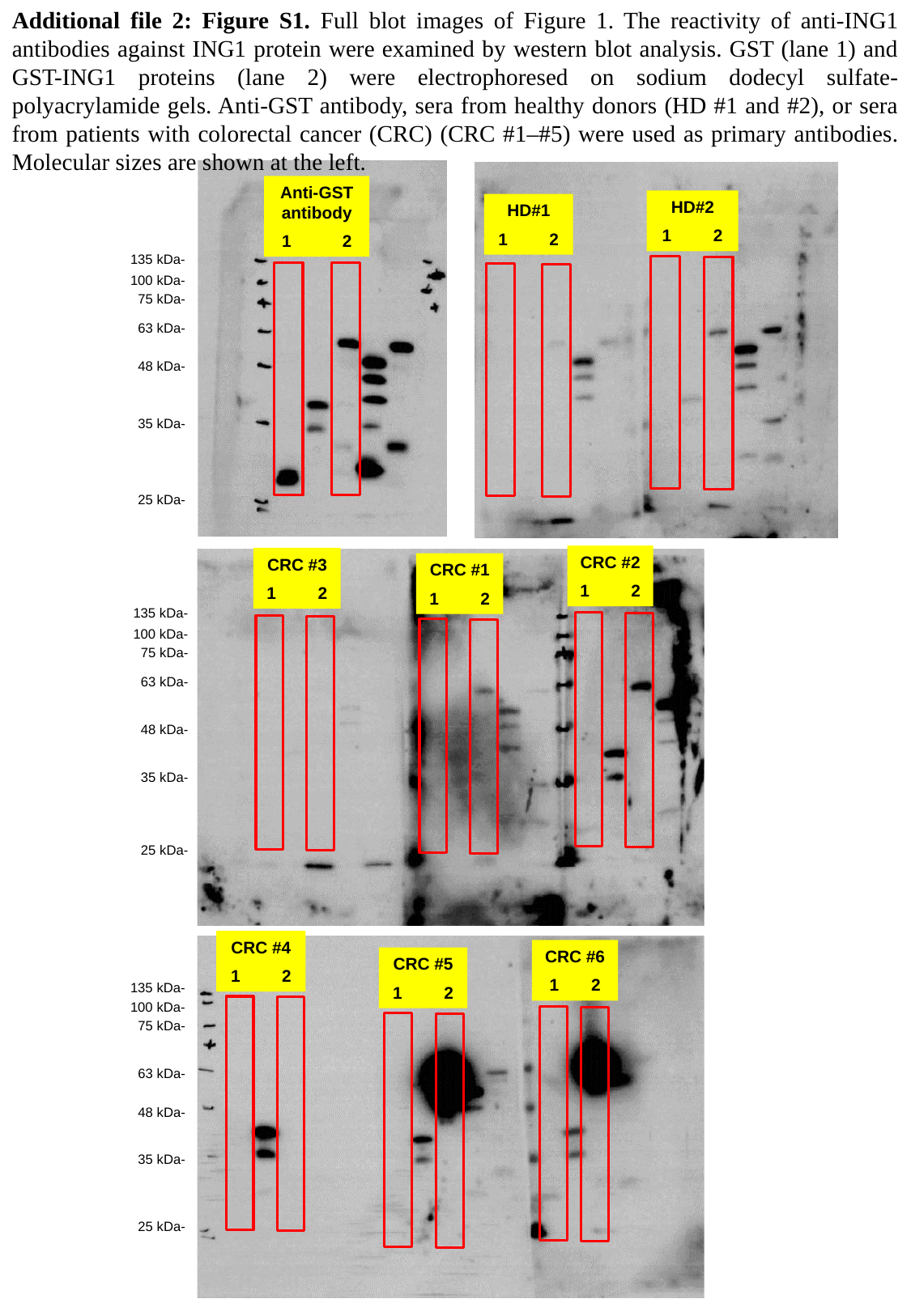

Additional file 2: Figure S1. Full blot images of Figure 1. The reactivity of anti-ING1 antibodies against ING1 protein were examined by western blot analysis. GST (lane 1) and GST-ING1 proteins (lane 2) were electrophoresed on sodium dodecyl sulfate-polyacrylamide gels. Anti-GST antibody, sera from healthy donors (HD #1 and #2), or sera from patients with colorectal cancer (CRC) (CRC #1–#5) were used as primary antibodies. Molecular sizes are shown at the left.
Anti-GST antibody
1 2
HD#2
1 2
HD#1
1 2
135 kDa-
100 kDa-
75 kDa-
63 kDa-
48 kDa-
35 kDa-
25 kDa-
CRC #2
1 2
CRC #3
1 2
CRC #1
1 2
135 kDa-
100 kDa-
75 kDa-
63 kDa-
48 kDa-
35 kDa-
25 kDa-
CRC #4
1 2
CRC #6
1 2
CRC #5
1 2
135 kDa-
100 kDa-
75 kDa-
63 kDa-
48 kDa-
35 kDa-
25 kDa-
